# Role of morphological traits and cell wall components in imparting resistance to pink stem borer, *Sesamia inferens* Walker in maize

**DOI:** 10.3389/fpls.2023.1167248

**Published:** 2023-07-24

**Authors:** P. Lakshmi Soujanya, J. C. Sekhar, Chikkappa G. Karjagi, C. V. Ratnavathi, R. Venkateswarlu, K. R. Yathish, S. B. Suby, N. Sunil, Sujay Rakshit

**Affiliations:** ^1^ Winter Nursery Centre, Indian Council of Agricultural Research (ICAR)-Indian Institute of Maize Research, Rajendranagar, Hyderabad, India; ^2^ Delhi Unit, Indian Council of Agricultural Research (ICAR)-Indian Institute of Maize Research, Pusa, New Delhi, India; ^3^ Plant Breeding, Indian Council of Agricultural Research (ICAR)-Indian Institute of Millets Research, Rajendranagar, Hyderabad, India; ^4^ Plant Breeding, Indian Council of Agricultural Research (ICAR)-Indian Institute of Agricultural Biotechnology Garkhatanga, Ranchi, India

**Keywords:** maize, host plant resistance, damage parameters, pink stem borer, antibiosis, biochemical constituents

## Abstract

Host Plant Resistance (HPR) is the most important component for sustainable management of insect pests. The purpose of the present work was to understand the role of various morphological and biochemical factors as defense mechanism and their interaction on different biological parameters attributed to survival and development of pink stem borer (PSB), *Sesamia inferens* Walker in maize. The resistant and moderately resistant genotypes (DMRE 63, CM 500 and WNZ Exotic pool) suffered least leaf injury rating (LIR), dead hearts (DH%), percentage stem tunneling (ST%), number of entry/exit holes (E/EH) and showed deleterious effects on biological parameters of pink stem borer as compared to susceptible ones (CM 202 and BML 6). Resistance index among the genotypes varied from 0.11 to 0.46. The variation in morphological traits such as number of nodes, internode distance and stem diameter could not distinguish all the resistant genotypes from that of susceptible genotypes in terms of its mean value. Higher levels of biochemical constituents, *viz*., *p*-Coumaric acid (*p*-CA), ferulic acid (FA), acid detergent fibre (ADF) and acid detergent lignin (ADL) were observed in resistant genotypes compared to susceptible ones. Antibiosis was expressed in terms of reduced pupal weight when fed on WNZ Exotic pool, whereas larval weight and larval survival affected when fed on DMRE 63. Higher concentration of *p*-CA content in pith of resistant maize genotypes prolonged the pupal period of pink stem borer. Higher concentration of *p*-CA and FA contents in rind reduced the adult emergence, as they showed significant negative correlation between them. The larval period was prolonged with higher levels of ADF and ADL contents in maize genotypes either in rind or both rind and pith as both ADF and ADL content showed a significant positive correlation with the larval period. The Pearson correlation analysis of most of the biochemical constituents revealed significant negative correlation with damage parameters. The correlation coefficients between *p*-CA with DH (%), ST (%) and E/EH were r= -0.9642^**^, r= -0.9363^**^, and r= -0.9646^**^, respectively. Similarly, the correlation coefficients between FA with DH (%), ST (%) and E/EH were r= -0.9217^*^, r= -0.9563^**^, and r= -0.9434^**^, respectively and ADF with DH (%), ST (%) and E/EH were r= -0.9506^**^, r= -0.9611^**^, and r= -0.9709^**^, respectively. The study confirms that stem damage parameters can also be used as selection criteria along with LIR to identify resistant genotypes against pink stem borer. Based on the correlation analysis it was concluded that resistance to pink stem borer in maize is the result of interaction of several morphological and biochemical traits rather than a single factor. The findings obtained from the present study can be utilised in pink stem borer resistance breeding programmes to enhance and diversify the basis of resistance.

## Introduction

Pink stem borer (PSB), *Sesamia inferens* Walker is the most important winter insect pest of maize in India, and is reported to cause 25.7-78.9% yield losses in maize (Rao, 1983). Though PSB infestation in maize commences at an early vegetative stage it can attack all stages of the plant. The first generation PSB feeds on foliage whereas second generation penetrate into maize stems and form galleries inside by feeding on pith. It is important to develop appropriate management strategies against PSB to reduce the yield losses in maize. Management of PSB through use of different chemicals has several challenges, especially with respect to its efficiency as larva feeds inside the stem. Apart from low efficiency, chemical control is also not economical for resource-poor smallholders and also has several concerns such as development of resistance, toxicity to natural enemies and environmental hazards. In such a scenario, host plant resistance (HPR) is one of the most promising approaches for PSB management which is economical and environment friendly. HPR can effectively complement other components of IPM to reduce the losses due to PSB damage in maize. In previous studies, variability in resistance to PSB has been observed among maize inbred lines based on Leaf Injury Rating (LIR) at the early whorl stage (Sarup et al., 1978; Sekhar et al., 2008; Sekhar et al., 2016; Lakshmi Soujanya et al., 2019; Lakshmi Soujanya et al., 2020). Apart from LIR, observations on stem tunneling is also been reported as one of the most important parameter which can be considered to identify the resistant genotypes against PSB (Malvar et al., 2008). Several authors have been used stem tunneling (%) as a major parameter to identify resistant genotypes in maize against African stem borer, *Sesamia calamistis* Hmps (Guthrie et al., 1971), southwestern corn borer *Diatrea grandiosella* Dyar (Davis and Williams, 1994) and European corn borer *Ostrinia nubilalis* Hubner (Bosque-Perez et al., 1989). Thus, apart from LIR, data on stem damage parameters provide additional information on the level of resistance of the genotypes and its effect on the yield reduction. Identification of resistant genotypes against PSB attack based on LIR and stem tunneling would be more effective to employ and integrate HPR to reduce the losses due to PSB attack.

Plants respond to insect attack by activating certain defense mechanisms. In general, the host plants exhibit morphological, physiological and biochemical changes at various phenological stages of the crop upon insect pest attack. The major morphological characters reported to impart resistance to insect pests are the presence of trichomes, the thickness of leaf, rind and pith, the length and number of internodes in maize (Kumar, 1997; Santiago et al., 2003; Santiago et al., 2011). The most common biochemical components which are reported to be associated with resistance reaction in plants against insect pests are sugars, amino acid, polyphenols and tannin (Kumar, 1997; Rao and Panwar, 2000). It was reported that the resistance reaction against PSB in maize is complex due to the involvement of several biochemical constituents (Lakshmi Soujanya et al., 2020). Cell wall hydroxycinnamic acids including *p*-Coumaric acid (*p*-CA), ferulic acid (FA) present in leaf and stem tissues are responsible for resistance to borers namely European corn borer (Bergvinson et al., 1997), Mediterranean corn borer (Santiago et al., 2008), Southwestern corn borer and sugarcane borer (Ramputh, 2002). Further, acid detergent fiber (ADF), acid detergent lignin (ADL) in maize leaf sheaths and stalks imparts resistance to stalk tunneling by the European corn borer (Coors, 1987). Hence, the selection for resistant genotypes against PSB in maize should be based on more than one parameter to make it more robust. Thus, the present study hypothesize that a more robust criteria for identification of resistant genotypes to PSB in maize is needed. Keeping in view of the above considerations, the present study was undertaken with the following objectives, namely, (i) to understand the morphological basis of PSB resistance (ii) to study the influence of the stalk tissues from different maize genotypes on the development of PSB and (iii) to determine the role of various biochemical traits such as *p*-Coumaric acid (p-CA), ferulic acid (FA), acid detergent fiber (ADF), acid detergent lignin (ADL) in PSB resistance.

## Materials and methods

### Experimental materials

Five maize inbred lines, *viz*., DMRE 63, CM 500, WNZ Exotic pool, CM 202 and BML 6 with known resistance response to PSB attack based on previous study were used (Lakshmi Soujanya et al., 2020). The details of the genotypes including their resistance responses were given in **Table 1**. The PSB larvae reared from the field population at Winter Nursery Centre, ICAR-Indian Institute of Maize Research (ICAR-IIMR), Hyderabad, Telangana, India was used for infestation in the present study.

**Table 1:** Stem damage parameters and associated morphological traits of maize genotypes.

Inbreds	Pedigree	Leaf Injury Rating (LIR) 1-9 Scale	Dead Hearts (DH)(%)	Stem tunneling (ST)(%)	Number of entry/exit holes (E/EH)	Selection Index	Number of nodes (NN)	Internode distance (ID) (cm)	Stem diameter (SD) (mm)
**DMRE 63**	CM 500 SEL	2.85 ± 0.10^d^	15.00 ± 10.0^b^	1.66 ± 0.10^c^	0.17 ± 0.10^b^	0.12	12.34 ± 0.13^d^	9.71 ± 0.33^c^	13.77 ± 0.27^d^
**CM 500**	Antigua Gr I	2.95 ± 0.11^d^	17.50 ± 5.0^b^	0.97 ± 0.10^d^	0.15 ± 0.10^b^	0.11	16.10 ± 0.29^a^	10.74 ± 0.31^b^	20.44 ± 0.52^a^
**WNZ Exotic Pool**	WNZPBTL1/2/3/4/5/6/7/8/9####	4.27 ± 0.10^c^	12.50 ± 5.0^b^	0.85 ± 0.1^d^	0.12 ± 0.10^b^	0.11	14.30 ± 0.14^c^	11.42 ± 0.27^a^	17.00 ± 0.52^c^
**CM 202**	C121E	7.85 ± 0.14^a^	37.50 ± 5.0^a^	9.10 ± 0.15^a^	1.22 ± 0.12^a^	0.45	14.65 ± 0.25^b^	11.16 ± 0.64^ab^	18.52 ± 0.53^b^
**BML 6**	SRRL 65-B96-1-1-2-#-2-2-1-⊗-1-1-⊗b-⊗b	7.15 ± 0.15^b^	42.50 ± 12.58^a^	8.50 ± 0.11^b^	1.37 ± 0.28^a^	0.46	14.85 ± 0.13^b^	11.36 ± 0.29^a^	18.92 ± 0.1^b^
**LSD (P=0.05)**		0.37	11.07	0.29	0.25		0.33	0.54	0.72

Means within a column followed by different letters are significantly different (LSD Test p = 0.05).

### Experimental design and treatments

The genotypes were evaluated for PSB attack under artificial infestation in the field experiment conducted in randomized complete block design (RCBD) with four replications. The experiment was carried out at Winter Nursery Centre (17.3254N; 78.4004E; 527 AMSL), ICAR-IIMR, Rajendranagar, Hyderabad, Telangana, India during November 2021 to March, 2022. The average day and night temperatures recorded were 26 ± 2°C and 15 ± 2°C, respectively. The plot size of each experimental unit was 7.5 m^2^ comprising four rows of 2.5 m in length with 75 × 20 cm spacing between rows and plants within a row, respectively. Sowing was done through dibbling with two seeds per hill and later thinned by retaining one plant per hill and a total of 48-52 plants per experimental unit were maintained. The crop was raised following recommended agronomic practices for inbred lines. The second-generation neonate larvae of PSB were released with the help of a camel hairbrush @ 10 larvae/plant on 12-day old seedlings. Observations on visual rating of leaf injury rating (LIR) on a 1-9 scale, number of dead hearts per plot at 45 DAG, stem damage *i*.*e*. tunnel length by splitting the main stem at harvest, and the number of entry/exit holes, after removing the leaf sheath at harvest were recorded on each genotype (Kumar, 1994). The resistant, moderately resistant, and susceptible lines are defined by LIR 1-3, 3.1-6 and 6.1-9, respectively (Reddy et al., 2003). 1- Apparently healthy plant; 2- Plant with parallel, oval or oblong holes, slightly bigger than pin sized (2-3 mm) on 1-2 leaves; 3- Plant with more elongated holes (4-5 mm or match stick head sized) or shot holes on 1-2 leaves; 4- Plant with injury (oval holes, shot holes and slits of 1-4 cm) in about 1/3 of total number of leaves and midrib damage on 1-2 leaves; 5- Plants with about 50% leaf damage, oblong holes, shot holes, slits and streaks of 5-10 cms and midrib damage on leaves; 6- Plants with a variety of leaf injuries to about two thirds of the total number of leaves (ragged appearance) or one or two holes or slits at the base of the stem (> 10 cms streaks are observed); 7- Plants with every type of leaf injury and almost all the leaves damaged (ragged or crimpled appearance), with tassel stalk boring or circular dark ring at the base of stem; 8- Plants with stunted growth in which all the leaves are damaged; 9- Plants with dead heart.

The data on additional parameters like number of nodes, internode distance and stem diameter were also recorded. The node above ground was considered as one and total number of nodes were measured in the whole plant. The length of internode was measured with the help of measuring tape whereas stem diameter was recorded with the help of vernier caliper on the internode below the top ear. The tunnel length was represented as percentage tunneling relative to plant height (Singh et al., 2011). These observations were recorded on ten randomly selected plants of each genotype in four replications and the average was calculated. The selection index based on leaf feeding damage score, dead-hearts (%), stem tunnel length (%) and number of entry/exit holes was constructed to determine the ranking of each line by summing up the ratios between values and overall mean and dividing by the number of parameters evaluated.

### Assessing antibiosis mechanisms of resistance by non-choice feeding bioassays

The genotypes subjected to field evaluation against PSB under artificial infestation were also evaluated for antibiosis component of resistance to PSB by following non-choice feeding bioassays under laboratory conditions at 27 ± 1 ^0^C and 60 **±** 5% relative humidity (RH). The basal part of the stalk tissues of each genotype upto second internode were collected from field in plastic jars and 20 neonate larvae were released. The experiment was laid out in a completely randomized design with five replications and the genotypes were grown separately in the field to collect the fresh stalk tissues. The stalk tissues in each plastic jar were replaced with freshly collected stalk tissues from field once in every two days until pupation. Observations namely mean larval weight (mg) at 10 days after release, larval period (days), pupal weight (mg), pupal period (days), percent adult emergence and adult longevity (days) were recorded on the number of larvae that survived at 10 days after release. The weights of larvae and pupae were taken with a precision analytical balance.

### Sampling for biochemical analysis

The genotypes (15 plants per genotype) were grown separately for biochemical analysis as well in plastic pots (30 cm in height, 30 cm top diameter and 19 cm bottom diameter), filled with black soil collected from the experimental field. The basal part of stem tissues (up to the second internode) above ground level was collected from three plants per replication of each genotype at the V_8_ phenological stage and manually separated into rind and pith tissues for biochemical analysis of these tissues separately. The stalk tissues were analysed in three replications. The samples were collected freshly and were immediately ground into fine powder in liquid nitrogen for extraction of various components.

### Extraction of selected hydroxycinnamic acids

A sample of 1000 mg of grounded rind and pith tissues was used for cell wall monomers *i.e. p-*Coumaric acid (*p*-CA) and Ferulic acid (FA) extraction. The fine powder of samples was mixed in 80 percent chilled ethanol (1:10 w/v) to extract phenolic acids. The contents were shaken in a rotary water bath for 20 minutes at room temperature. The samples were then centrifuged at 5000 rpm for 5 minutes and the resulting pellets containing bound phenolic acids were stored at -40°C until alkaline hydrolysis. Since some soluble phenols can be present in the pellets, the pellet/residue was re-extracted twice with 10 ml of 80% chilled ethanol before proceeding for alkaline hydrolysis. Later, the pellet was transferred into a tube and 10 ml of 4 N sodium hydroxide was added. The mixture was flushed with nitrogen gas for 30 seconds to create an inert environment. The flask containing an alkaline hydrolyzed mixture was sealed and kept for shaking on a rotary water bath at 65°C for 90 minutes. After alkaline hydrolysis, the mixture was acidified to pH 2 with 6 N HCl. Subsequently, the mixture was centrifuged at 5000 rpm for 5 minutes, to remove the flocculated material. Then, the acidified bound phenolic acids were extracted five times in n-hexane (1:1 v/v) and the upper layer was discarded. Further, the residue was extracted with ethyl acetate six times. Ethyl acetate fractions were combined, evaporated to dryness under a water bath, reconstituted in 5 ml of 70 percent methanol, and stored at -40°C for analysis (Hung et al., 2009).

### Determination of p-CA and FA contents

The *p*-CA and FA content were determined using Shimadzu Ultra-Fast Liquid Chromatography (UFLC) equipped with an SPD-M20A Prominence photodiode array detector. The HPLC pumps, autosampler, column temperature and diode array system were monitored and controlled using the LC Solution Chromatography data software program. *p*-CA and FA separation were performed on the C18-Phenomenex column (250 × 4.6 mm). The column was held at 35°C and the flow rate was set at 1.0 ml per minute. The solvent system consisted of 2 percent glacial acetic acid (A) and 100 percent Acetonitrile (B). A gradient program of 15 percent B and 85 percent A for 20 minutes was followed by a 20 µl sample injection. The analysis was done by preparing samples in three replications. The peaks of *p*-CA and FA were identified by standards at 325 nm with retention times of 10.9 and 11.6 minutes, respectively. The amount of *p*-CA and FA in maize stalk samples was quantified by the calibration curve of respective standards with the help of LC Solution Software.

### Determination of acid-detergent fiber and acid-detergent lignin

The estimation of ADF and ADL components was carried out in collected tissues using AOAC official method 973.18: Fibre (Acid detergent) and lignin (H_2_S0_4_) in animal feed (AOAC Official Method, 1997). The determination of ADF and ADL was performed using Fiber Bag system (Fiberthem, C. Gerhardt, Germany) as per manufacturer instructions.

### Statistical analysis

The data were subjected to analysis of variance (ANOVA) by using a general linear model PROC GLM (SAS Institute, 2011). The significant differences between the genotypes were tested by *F*-tests and the treatment means were judged by using the least significant differences (LSD). Further, the data of **Tables 1**, **2** were subjected to Pearson correlation using PROC CORR, a procedure of SAS, to understand the association among various plant characteristics and biochemical constituents with biological parameters of PSB. All the analyses were performed in SAS version 9.3.

**Table 2:** Biochemical constituents in rind and pith tissues of maize genotypes at V8 phenological stage.

	*p*-CA (mg/g)		FA (mg/g)		ADF (%)		ADL (%)	
	Rind	Pith	Rind	Pith	Rind	Pith	Rind	Pith
**DMRE 63**	1.48 ± 0.09^ab^	0.63 ± 0.03^b^	0.88 ± 0.01^b^	0.47 ± 0.02^c^	42.30 ± 1.23^a^	23.10 ± 0.29^cd^	6.21 ± 0.85^a^	4.39 ± 0.25^a^
**CM 500**	1.62 ± 0.01^a^	0.84 ± 0.10^a^	1.06 ± 0.01^a^	0.54 ± 0.03^b^	41.22 ± 0.94^ab^	27.35 ± 1.0^b^	4.21 ± 0.27^b^	2.65 ± 0.1^b^
**WNZ Exotic Pool**	1.65 ± 0.13^a^	0.75 ± 0.01^a^	1.04 ± 0.01^a^	0.64 ± 0.01^a^	39.78 ± 1.11^b^	33.65 ± 0.94^a^	4.60 ± 0.46^b^	2.14 ± 0.31^b^
**CM 202**	1.15 ± 0.38^bc^	0.59 ± 0.01^b^	0.66 ± 0.06^c^	0.44 ± 0.01^cd^	33.31 ± 0.94^c^	23.71 ± 1.32^c^	3.30 ± 1.08^b^	1.90 ± 0.65^b^
**BML 6**	0.89 ± 0.09^c^	0.56 ± 0.02^b^	0.64 ± 0.01^c^	0.42 ± 0.01^d^	33.11 ± 1.37^c^	20.94 ± 1.72^d^	3.26 ± 0.12^b^	1.94 ± 0.49^b^
**LSD**	0.36	0.09	0.05	0.04	1.77	2.17	1.37	0.75

Means within a column followed by different letters are significantly different (LSD Test p = 0.05).

## Results

### Variation in morphological parameters vis-à-vis PSB infestation

The genotypes which were characterized previously against PSB infestation were used in the present study to generate data on additional parameters which are more relevant to corroborate the resistance response of the genotypes against PSB. Four major damage parameters namely leaf injury rating (LIR), per cent dead hearts (DH%), per cent stem tunneling (ST%) and the number of entry/exit holes (E/EH), which are directly related to resistance response of the genotypes showed significant differences in the mean values of resistant and susceptible genotypes (**Table 1**). For example, DMRE 63 (2.85) and CM 500 (2.95) were resistant as minimum LIR was recorded. WNZ Exotic pool (4.27) was considered as moderately resistant. CM 202 (7.85) and BML 6 (7.15) were susceptible to PSB with LIR score of >7. The mean value of DH (%) of resistant genotypes, *viz*., DMRE 63 (15.00%), CM 500 (17.50%) and WNZ Exotic pool (12.50%) were significantly lower as compared to susceptible genotypes. Similarly, resistant genotypes recorded significantly lower mean values of ST (%) and E/EH compared to susceptible ones. The mean value of ST% and E/EH in resistant genotypes, *viz*., WNZ Exotic pool, CM 500 and DMRE 63 were 0.85%, and 0.12, 0.97%, and 0.15, and 1.66%, and 0.17 respectively, whereas in susceptible genotypes it were comparatively lower. The resistance index (RI) among genotypes varied from 0.11 to 0.46. Lowest RI was observed in CM 500, WNZ Exotic pool, DMRE 63 whereas highest RI was recorded in CM 202 (0.45) and BML 6 (0.46).

In a similar way, the mean value of other traits like number of nodes (NN), internode distance (ID) and stem diameter (SD) were significantly different among the genotypes. Highest NN were recorded in the genotype CM 500 (16.10) while the lowest was observed in DMRE 63 (12.34). The highest ID was observed in WNZ Exotic pool (11.42 cm) followed by BML 6 (11.36 cm) and CM 202 (11.16 cm), while the lowest ID was observed in DMRE 63 (9.71 cm). The highest and lowest SD were recorded in genotypes CM 500 (20.44 mm) and DMRE 63 (13.77 mm), respectively.

### Assessment of antibiosis

**Figure 1:**
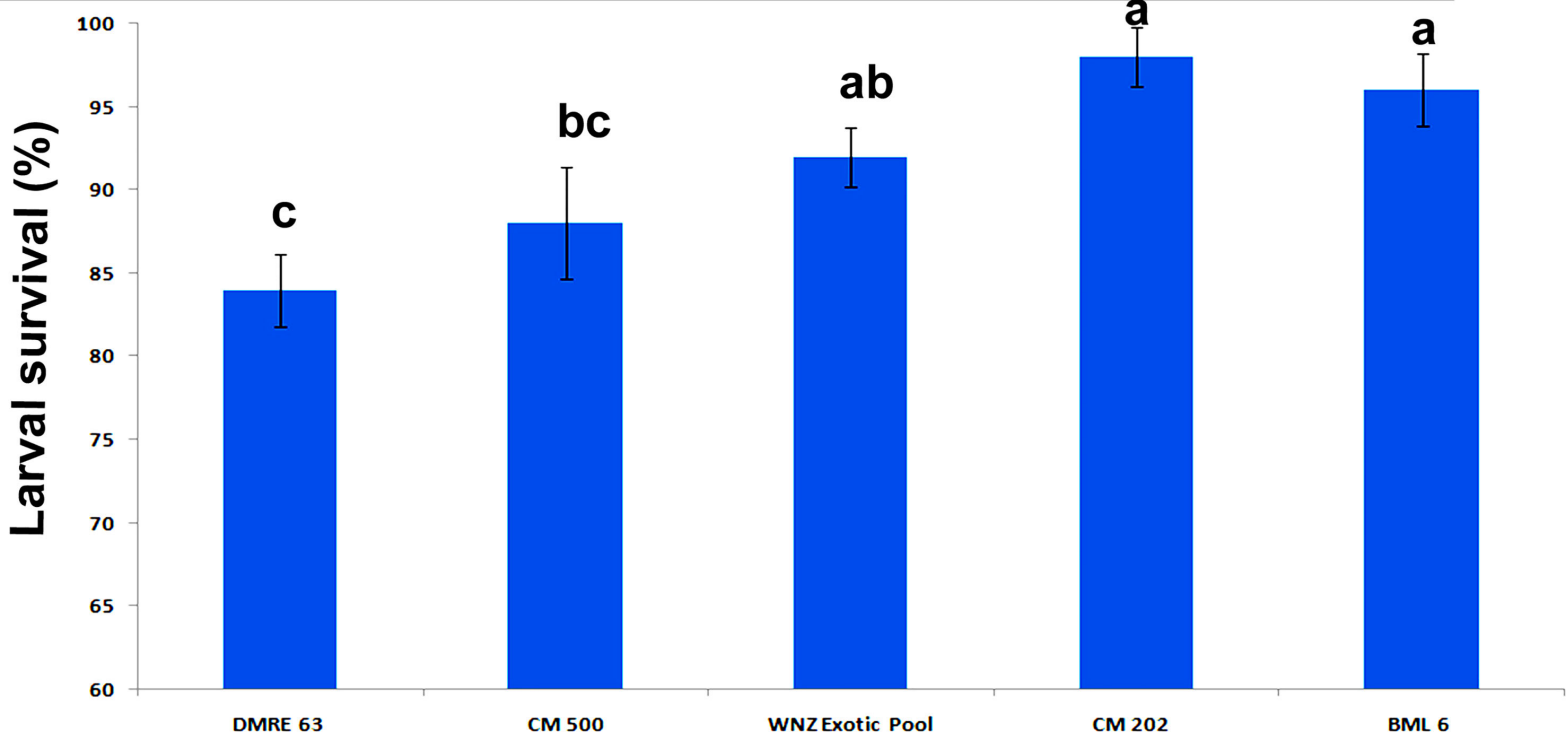
Larval survival (%) of pink stem borer in different maize genotypes. Error bar indicates mean ± SEM of five replications. Different letters represent statistically significant differences determined by one-way ANOVA (ρ < 0.05).

**Figure 2:**
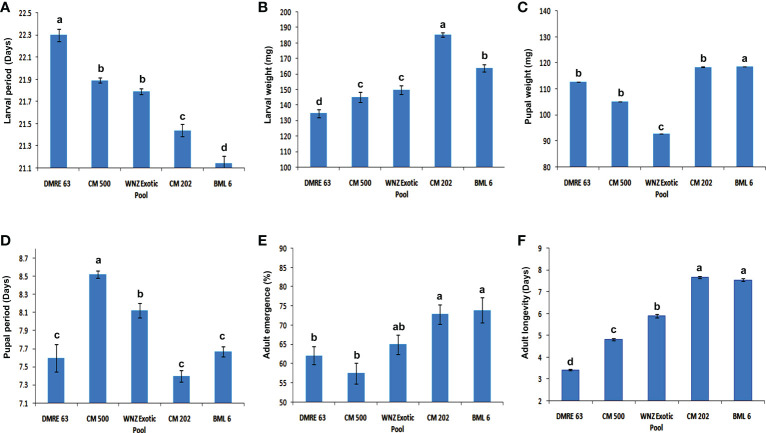
Reaction of maize genotypes to pink stem borer **(A)** Effect on larval period (days) in different maize genotypes. **(B)** Effect on larval weight (mg) in different maize genotypes. **(C)** Effect on pupal weight (mg) in different maize genotypes. **(D)** Effect on pupal period (days) in different maize genotypes. **(E)** Effect on adult emergence (%) in different maize genotypes. **(F)** Effect on adult longevity (days) in different maize genotypes. Each treatment was replicated five times. The experimental data were analyzed using one-way analysis of varience and the means (N=5) were compared using LSD test. ^*^P<0.05. Error bar indicates mean ± SEM of five replications. Different letters represent statistically significant differences determined by one-way ANOVA (ρ < 0.05).

The ANOVA showed significant genotypic differences for different biological parameters of PSB. With respect to the larval survival rate, the resistant genotypes were clearly distinguished from susceptible genotypes (**Figure 1**). The larval survival rate was significantly higher when fed on two susceptible genotypes BML 6 (96.00%) and CM 202 (98.00%) whereas in resistant genotypes it was observed as 84.00% in DMRE 63 and 88.00% in CM 500. Similarly, significant differences in the larval period and larval weight were also observed between resistant and susceptible genotypes (**Figures 2A, B**). The larval period was significantly longer when fed on one of the resistant parents, DMRE 63 (22.30 days) as compared to other genotypes like CM 500 (21.89 days) and WNZ Exotic pool (21.79). Significantly, lowest larval weights were observed when PSB fed on resistant genotypes namely DMRE 63 (134.68 mg), CM 500 (145.22 mg) and WNZ Exotic pool (149.87 mg) as compared to susceptible genotypes while significant lower pupal weight was observed in one of the resistant genotypes WNZ Exotic pool (92.70 mg) as compared to other genotypes (**Figure 2C**). The pupal period was significantly longer on two of the resistant genotypes namely CM 500 (8.52 days) and WNZ Exotic pool (8.12 days) as compared to the remaining genotypes (**Figure 2D**). Similarly, adult emergence was significantly lower from two of the resistant genotypes namely DMRE 63 (62.14%) and CM 500 (57.50%) (**Figure 2E**). The adults emerged from the susceptible genotypes, *viz.*, BML 6 (7.55 days) and CM 202 (7.67 days) survived longer when compared to the adults emerged from resistant genotypes (**Figure 2F**).

### Biochemical constituents in the basal part of stem tissues

The base of the plant is the first entry point of PSB; therefore, genotypes were analyzed for the biochemical constitution at base of the stalk. The results showed that the contents of all the four compounds (*p*-CA, FA, ADF and ADL) in rind and pith tissues differ significantly among genotypes at this stage (**Table 2**). The resistant genotypes namely CM 500 (1.62 mg/g and 0.84 mg/g) and WNZ Exotic pool (1.65 mg/g and 0.75 mg/g) showed significantly higher *p-*CA content both in rind and pith, whereas in susceptible genotypes it was significantly lower *i.e.* 0.89 mg/g, and 0.56 mg/g in BML 6 and 1.15 mg/g, and 0.59 mg/g in CM 202. The accumulated content of *p*-CA was 1.55 and 1.28-fold higher in resistant genotypes as compared to susceptible ones in rind and pith tissues, respectively. Two of the three resistant genotypes namely CM 500 (1.06 mg/g, 0.54 mg/g) and WNZ Exotic pool (1.04 mg/g, 0.64 mg/g) showed significantly higher level of FA in both rind and pith, respectively as compared to susceptible genotypes. The FA content observed in DMRE 63 was 0.88 mg/g, and 0.47 mg/g, in rind and pith tissues, respectively. In resistant genotypes, the accumulated content of FA was 1.52 and 1.27-fold higher in rind and pith, respectively when compared to susceptible ones. The ADF content in rind and pith of resistant and moderately resistant genotypes (DMRE 63, CM 500 and WNZ Exotic pool) were significantly higher and varied from 39.78% to 42.30% and 23.10 to 33.65%, respectively. The susceptible genotypes showed significantly lower ADF content in rind and pith, respectively. However, the accumulated ADF content was 1.23 and 1.25-fold higher in resistant genotypes as compared to susceptible ones in rind and pith, respectively.

On the contrary, resistant genotype DMRE 63 showed significant difference in ADL content in both rind (6.21%) and pith (4.39%) as compared to susceptible genotypes. The ADL content in rind and pith of CM 500 were 4.21% and 2.61% whereas in WNZ Exotic pool it was recorded as 4.60% and 2.14%, respectively. In susceptible genotypes, ADL content in rind was 3.26% in BML 6 and 3.30% in CM 202 whereas in pith it was 1.90% and 1.94% in CM 202 and BML 6, respectively. More notably, in resistant genotypes, the accumulated content of ADL was 1.52 and 1.59-fold higher when compared to susceptible ones in rind and pith, respectively. In general, the content of all the four biochemical compounds were relatively much higher in rind as compared to pith across all the genotypes.

### Correlation of morphological, damage parameters and biochemical constituents with different biological attributes of PSB

The Pearson correlation analysis among damage and morphological parameters in different maize genotypes were presented in **Table 3**. The LIR value in different maize genotypes showed significant and positive correlation with DH (%) (r=0.9124^*^), ST (%) (r=0.9563^**^) and E/EH (r=0.9474^**^). The DH (%) were also significantly positively correlated with ST(%) (r=0.9746**) and E/EH (r= 0.9926**).The correlation coefficients between ST (%) and E/EH (r=0.9896^**^), SD and NN (r=0.9861**) were also found significant and positive (**Table 4**). However, none of the morphological parameters showed significant association with LIR, DH (%), ST (%) and E/EH.

**Table 3:** Pearson’s correlation coefficients among damage and morphological parameters in different maize genotypes.

Parameters	LIR (1-9 Scale)	Dead Hearts (DH)(%)	Stem tunneling (ST)(%)	Number of entry/exit holes (E/EH)	Number of nodes (NN)	Internode distance (ID)	Stem diameter (SD)
**LIR (1-9 Scale)**	1						
**Dead Hearts (DH)(%)**	0.9124^*^	1					
**Stem tunneling (ST)(%)**	0.9563^**^	0.9746^**^	1				
**Number of entry/exit holes (E/EH)**	0.9474^**^	0.9926^**^	0.9896^**^	1			
**Number of nodes (NN)**	0.2107	0.2654	0.1414	0.1947	1		
**Internode distance (ID)**	0.6372	0.4559	0.4262	0.4773	0.6269	1	
**Stem diameter (SD)**	0.3493	0.4195	0.2996	0.3505	0.9861^**^	0.6519	1

*Correlation coefficients significant at P=0.05; **Correlation coefficients significant at P=0.01.

**Table 4:** Pearson’s correlation coefficients for various morphological, stem damage parameters and biochemical constituents with biological attributes of PSB in different maize genotypes.

Morphological/stem damage/biochemical parameters	Larval survival	Larval period	Larval weight	Pupal period	Pupal weight	Adult emergence	Adult longevity
**Number of nodes (NN)**	0.4197	-0.5154	0.3486	0.5722	-0.1081	-0.0311	0.4523
**Internode distance (ID)**	0.8367	-0.8176	0.6361	0.0916	-0.1763	0.5495	0.8480
**Stem diameter (SD)**	0.5235	-0.6244	0.4655	0.4454	0.0447	0.1099	0.5585
**Dead Hearts (DH)**	0.7996	-0.8759*	0.8027	-0.5990	0.7929	0.8669^*^	0.8305
**Stem tunneling (ST)(%)**	0.8298	-0.8225	0.8729*	-0.7236	0.7933	0.9165*	0.8381
**Number of entry/exit holes (E/EH)**	0.8331	-0.8755*	0.8335	-0.6641	0.7700	0.9187*	0.8556
p-Coumaric acid (p-CA)							
**In rind**	-0.6840	0.8002	-0.6609	0.6947	-0.8228	-0.8803*	-0.7243
**In pith**	-0.4721	0.4687	-0.5080	0.9538**	-0.7707	-0.8459	-0.4767
Ferulic acid (FA)							
**In rind**	-0.6629	0.6795	-0.7183	0.8580	-0.8731*	-0.8979*	-0.6749
**In pith**	-0.2905	0.3938	-0.4331	0.7096	-0.9964**	-0.5609	-0.3180
Acid Detergent Fibre (ADF)							
**In rind**	-0.9404**	0.9352**	-0.9069*	0.5844	-0.6058	-0.9395**	-0.9527**
**In pith**	-0.1461	0.3000	-0.2737	0.6381	-0.9681**	-0.4525	-0.1853
Acid Detergent Lignin (ADL)							
**In rind**	-0.9059*	0.9341*	-0.8369	0.0959	-0.3183	-0.6492	-0.9274*
**In pith**	-0.8944*	0.8631*	-0.7550	-0.0446	0.024	-0.5772	-0.9018*

*Correlation coefficients significant at P=0.05; **Correlation coefficients significant at P=0.01.

The correlation coefficients were also calculated among different biological parameters of PSB and morphological, damage attributes of genotypes as well as different biochemical compounds to assess the antibiosis component of resistance. DH (%), ST (%) and E/EH showed correlation with one or two biological parameters of PSB larvae. For example, DH (%) showed significantly negative correlation with larval period (r= -0.8759^*^) and positively correlated with adult emergence (r=0.8669^*^) while ST (%) showed significantly positive correlation with larval weight (r= 0.8729^*^) and adult emergence (r= 0.9165^*^) (**Table 4**). The E/EH showed significantly negative correlation with larval period (r=-0.8755^*^) while significant positive correlation with adult emergence (r= 0.9187^*^) was observed.

A significant negative correlation was observed between *p*-CA content in rind and adult emergence (r= -0.8803^*^). However, the correlation between *p*-CA content in pith and pupal period (r= 0.9538^**^) was significant and positive while non-significant negative correlation was found with adult emergence (r=-0.8459). Further, a non-significant positive correlation was observed between *p*-CA content in rind tissues and larval period (r=0.8002) whereas with pupal weight a negative correlation was observed (r=-0.8228). Similarly, the correlations between FA, ADF and ADL contents and different biological parameters of PSB were observed. The FA content in rind and pith tissues showed significant negative correlation with pupal weight (r= -0.8731^*^ with rind and r= -0.9964^**^ with pith). Similarly, the FA content in rind showed significant negative correlation with adult emergence (r= -0.8979^*^). The ADF content in rind tissues showed significant positive correlation with larval period (r= 0.9352^**^) and significant negative correlation with larval survival (r= -0.9404^**^), larval weight (r=-0.9069^*^), adult emergence (r= -0.9395^**^), adult longevity (r= -0.9527^**^); while ADF in pith showed significant negative correlation with pupal weight (r= -0.9681^**^). A significant positive correlation was observed between ADL content in rind and pith and larval period (r= 0.9341^*^, r= 0.8631^*^) while significant negative association was observed between ADL content in rind and pith with larval survival (r= -0.9059^*^, r= -0.8944^*^) and adult longevity (r= -0.9274^*^, r= -0.9018^*^).

The correlation coefficients were also calculated between different biochemical constituents with various damage and morphological attributes determining resistance against PSB (**Table 5**). A significant negative correlation was observed between *p*-CA, FA and ADF contents in rind tissues with DH %, ST (%) and E/EH and the correlation coefficients between *p*-CA with DH (%), ST (%) and E/EH were r= -0.9642^**^, r= -0.9363^**^, and r= -0.9646^**^, respectively. Similarly, the correlation coefficients between FA with DH (%), ST (%) and E/EH were r= -0.9217^*^, r= -0.9563^**^, and r= -0.9434^**^, respectively and ADF with DH (%), ST (%) and E/EH were r= -0.9506^**^, r= -0.9611^**^, and r= -0.9709^**^, respectively. The ADL content in pith tissues showed significant negative correlation with ID (r= -0.9738^**^).

**Table 5:** Pearson’s correlation coefficients among morphological, stem damage parameters with biochemical constituents in different maize genotypes.

Parameters	*p*-Coumaric acid (p-CA)		Ferulic acid (FA)		Acid Detergent Fibre (ADF)		Acid Detergent Lignin (ADL)	
	Rind	Pith	Rind	Pith	Rind	Pith	Rind	Pith
**Number of nodes (NN)**	-0.054	0.4579	0.1216	0.1320	-0.2883	0.1739	-0.7371	-0.7108
**Internode distance (ID)**	-0.3289	-0.0270	-0.2046	0.1976	-0.6572	0.3033	-0.8340	-0.9738**
**Stem diameter (SD)**	-0.2129	0.3080	-0.0430	-0.0194	-0.4282	0.0342	-0.8232	-0.7544
**Dead Hearts (DH)**	-0.9642**	-0.7330	-0.9217*	-0.7821	-0.9506**	-0.6990	-0.7868	-0.5657
**Stem tunneling (ST) (%)**	-0.9363**	-0.7998	-0.9563**	-0.7708	-0.9611**	-0.6638	-0.7365	-0.5387
**Number of entry/exit holes (E/EH)**	-0.9646**	-0.7814	-0.9434**	-0.7566	-0.9709**	-0.6628	-0.7696	-0.5751

*Correlation coefficients significant at P=0.05; **Correlation coefficients significant at P=0.01.

## Discussion

The present study demonstrates that it is not one or two traits or parameters, be it morphological attributes or biochemical compounds, but the different combinations of the parameters determine the overall response of the host plant against PSB. The responses of the genotypes against PSB attack were characterized earlier in our previous studies based on LIR (Leaf Injury Rating) in 1-9 scale (Lakshmi Soujanya et al., 2020). The resistant, moderately resistant, and susceptible lines are defined by LIR 1-3, >3.1-6 and >6.1-9, respectively. Apart from the reported ones including DMRE 63, CM 500, another genotype CM 111 *Zea diploperrenis* CM 111 (MIL1-11) was also found resistant to PSB in which LIR observed was 2.92 based on five seasons data at Hyderabad location. However, in the present study, it has been assumed that the stem damage by PSB is also important along with LIR as it accounts to provide protection from second generation PSB also. In order to elucidate further and corroborate resistance reaction of the genotypes against PSB under artificial infested conditions, the data on several additional parameters comprising various morphological and biochemical parameters from host side and biological parameters of PSB are generated. The extent of damage was significantly lower in resistant genotypes as compared to susceptible genotypes which might be due to the inability of PSB larva to make extensive leaf damage and long tunnels inside the stem in resistant genotypes. The genotypes that had lower LIR value also had lower DH (%), ST (%) and E/EH and vice versa. The findings of this investigation showed, that along with LIR, stem damage parameters can determine the resistance reaction of the genotypes and also affect indirectly some of the biological parameters of PSB. Selection Index was also formulated based on LIR score, DH (%), ST (%) and E/EH which provides efficiency in improvement of pink stem borer resistance in maize. However, the variation in other morphological traits cannot distinguish all the resistant genotypes from that of susceptible genotypes in terms of its mean value.

Information on mechanisms of resistance and its related factors is necessary for the effective utilization of resistant sources in the breeding programs. It is reported that quality of host plant determines the antibiotic resistance in plants (Dhillon and Chaudhary, 2015). In general, antibiotic resistance is tissue-specific and the area of the plant attacked by insect pests is crucial to determine the mechanisms of antibiotic resistance (Ordas et al., 2002). In the current research, data was generated on the biochemical compounds present in the basal part of stem tissue to assess the presence of antibiotic resistance and also chemical compounds which impart antibiotic resistance to host plants. The antibiosis study indicates that the survival and development of PSB greatly affected when PSB larvae fed on resistant genotypes as compared to susceptible ones. It can be assumed that the factors which affect larval weight also affected other biological parameters such as larval survival, pupal weight, adult emergence and adult longevity of PSB. Therefore, evidence is building up that the chemical compounds affected the different biological parameters of PSB but the degree varied between compounds. Earlier studies showed that antibiosis results in decreased larval weight and longer larval development of stem borers when fed on different maize genotypes with varying levels of resistance (Kumar et al., 2006; Dhillon and Chaudhary, 2015).

Furthermore, it is important to consider the fact that the point of attack by PSB is lower part of the stem. As rind is the first point of contact between PSB and host plant, rind acts as first mechanical barrier against PSB attack. Therefore, biochemical constituents in rind has not only differed significantly in the mean values between resistant and susceptible genotypes, but also showed better correlations with biological parameters of PSB. The pith is the main tissue on which PSB feeds after its entry into the stem. Pith antibiosis can also act as one of the factors that confer stem resistance against PSB attack. In the present study, pith antibiosis is evident in all the resistant genotypes. For example, CM 500 and WNZ Exotic pool due to presence of *p*-CA, FA and ADF contents and DMRE 63 due to ADL presence in pith, showed an antibiotic pith that affected one or the other biological attributes of PSB. Antibiosis is expressed in terms of reduced pupal weight when fed on WNZ Exotic pool, whereas larval weight and survival affected when fed on DMRE 63. This is because larva may have consumed less stem tissues showing growth reduction and lowest larval survival when fed on these genotypes. In a similar way, antibiosis property of pith is also reported when maize inbred lines namely CM 151, CO 125 and EP 139 with different levels of stem resistance was evaluated in maize against *Sesamia nonagrioides* (Ordas et al., 2002). The result conferred that pith antibiotic compound imparted stem resistance along with other possible defense mechanisms. In our previous studies, biochemical traits such as *p*-CA and FA were analysed in leaf and whole stem tissues of maize to understand the resistance response of the genotypes against PSB (Lakshmi Soujanya et al., 2020). However, in the present study, it is hypothesized that the basal part of stem tissues of maize genotypes up to the second internode impart resistance as this is the initial entry point of the PSB larva into the maize plant and also PSB feed on it in the establishment period. The results in the present study indicated that the presence of different biochemical constituents, their combinations and levels of expression together affected different biological parameters of PSB.

The findings of the current research agree with other researchers who observed significant variation in maize inbred lines with respect to different biochemical compounds (Huang et al., 2013) and concluded that hydroxycinnamate concentrations are associated with increased stem resistance to Mediterraneancorn borer (Santiago et al., 2016; Gesteiro et al., 2021) and European corn borer (Bergvinson et al., 1997). Barros-Rios et al. (2011) measured cell wall bound forms of hydroxycinnamic acids including the total ester and ether linked FA monomers in rind and pith tissues of maize. The ester bound p-CA was the most abundant hydroxycinnamic acid detected in maize pith (2.1%) and rind tissues (2.7%) of total cell wall followed by ester plus ether bound FA in pith (1.4%) and rind (1.5%) of total cell wall; together accounted less than the 5% of the total cell wall in both tissues. Significantly higher lignin, FA ethers, 8-O-4-DFA, *p*-CA, and total cell wall material was found in resistant line EP 39 compared to susceptible ones. The ADF and ADL contents in maize have also been reported to be associated with resistance to stalk tunneling by the European corn borer (Coors, 1987; Beeghly et al., 1997; Cardinal et al., 2001; Cardinal et al., 2003; Santiago and Malvar, 2010). In particular, it was reported that high S subunit-enriched lignin content is associated with stem strength and resistance to biotic stresses (Zheng et al., 2017; Ma et al., 2018). Ostrander and Coors (1997) observed significant positive linear relationship between second generation ECB resistance and ADF and lignin in the stalk tissue of BS9 (CB) cycles of selection. Cardinal and Lee (2005) analysed ADF, ADL contents to know the relationship between resistance to European corn borer and cell wall components in maize. The results revealed that ADF, ADL contents present in leaf sheath and stalk tissues contribute to European corn borer resistance in a maize recombinant inbred line population derived from inbred lines B73 and B52. Notably, there is disparity in concentrations of biochemical contents which were much higher in the rind as compared to pith tissues in the tested maize genotypes. This is in agreement with the conclusion reported by Santiago et al. (2011) who observed increased concentrations of *p*-CA, FA, ADF and ADL contents in rind as compared to pith tissues of maize genotypes. This is because rind vascular tissues lignify to a greater extent to support the conductive and supportive tissues of the internode (Morrison et al., 1998). Lignins, the primarily syringil units are acylated at the gamma position by p-coumarates and most *p*-CA accretion occurs in tandem with lignifications (Lu and Ralph, 1999). Therefore, a higher accumulation of *p-*CA is an indicator for lignin deposition which is directly related to corn borer and other biotic stresses resistance (Zhao et al., 2010; Santiago et al., 2016; Gesteiro et al., 2021).

Apart from *p*-CA, FA, ADF and ADL compounds, there are reports of other biochemical constitutents which impart resistace to stem borers in maize. Hydroxamic acid- (2,4, dihydroxy-7-methoxy- -(2H)-1,4-benzoxazin-3-(4H)-one (DIMBOA) inhibits feeding by European corn borer (Klun and Robinson, 1969) and Mediterranean corn borer (Ortego et al., 1998) at early stages of maize. AS DIMBOA concentration decreases as the plant grows, it cannot provide protection to second generation stem borers (Mihm, 1985). A positive correlation was observed between high total diferulate content in different maize genotypes and decreased Mediterranean corn borer damage (Santiago et al., 2008).

The correlations among damage parameters revealed strong significant positive association of LIR with other damage parameters which proved as visual indicators of PSB resistance. Earlier, Anil et al. (2018) reported significant positive correlation between LIR and ST (%) caused by *Chilo partellus* in maize. The correlation study between biological parameters of PSB and different damage parameters indicated that DH (%), ST (%), and E/EH showed significant negative correlation with larval period, while few damage parameters like ST (%) with larval weight and ST (%), DH (%), and E/EH with adult emergence showed significant positive correlation. Also, there is a positive correlation among DH (%), ST (%) and E/EH with larval survival and adult longevity, but non-significant. The results from correlation studies suggest that damage parameters and insect biological attributes are important resistance determining factors against PSB in maize.

Higher concentration of *p*-CA content in pith of maize genotypes prolongs the pupal period of PSB as they showed significant positive correlation between them. On the contrary higher concentration of *p*-CA and FA contents in rind reduce adult emergence, as they showed significant negative correlation between them. Furthermore, present result indicated that the higher levels of FA content in rind and pith tissues reduce the pupal weight of PSB; pupal weight showed significant negative correlation with FA content in rind and pith of maize genotypes. ADF and ADL being complex compounds, do not serve as energy source; it is evident in the present study that the larval period prolongs with higher levels of these fiber contents in maize genotypes either in rind (ADF) or both rind and pith (ADL) as both ADF and ADL content showed a significant positive correlation with the larval period. The present result is in agreement with Hedin et al. (1984) who reported that genotypes resistant to South Western corn borer contained high amount of crude fibre content compared to susceptible ones. On the contrary a significant negative association between ADF content in rind and larval survival, larval weight, adult emergence and adult longevity; and ADF content in pith with pupal weight; and ADL content in rind and pith with larval survival and adult longevity, suggest that these biochemical constituents together affect the overall survival and development of PSB and play a significant role in imparting resistance to PSB. The presence of biochemical compounds namely *p*-CA, FA and ADF in rind acts as an important component in imparting resistance against PSB. These compounds showed significant negative correlation with damage parameters. The results indicate that the presence of these compounds in higher concentration in rind tissues minimizes the damage by PSB. For instance, Gesteiro et al. (2021) in a similar study also found significant negative correlation between *p*-coumaric acid and stem tunnel length, caused by *Sesamia nonagrioides* larvae. The present study observed significant variation for morphological, stem damage parameters and biochemical traits and also the significant association between these parameters. The results of the present study corroborates with Dhillon and Chaudhary (2018) who reported that the interaction of several morphological, and biochemical constituents impart resistance to stem borers rather than a single component.

In conclusion, the present study showed that similar to LIR, DH (%), ST (%) and E/EH are directly related to the resistant and susceptible response of the genotypes. Thus, the stem damage parameters can also be used as selection criteria along with LIR to develop resistant genotypes against PSB. However, it may be labour intensive for large scale screening as the data on ST (%) and E/EH to be taken at the time of harvest. Stem damage parameters can be considered as an additional information to further study the mechanism of resistance. One of the most important traits which is involved in determining resistance is the structural reinforcement of cell wall at the basal part of the internode as it is the initial entry point of PSB. Targeting stem resistance in breeding programs is advantageous as it protects the damage from two generations of PSB and also good standability ensures good harvest.

## Data availability statement

The original contributions presented in the study are included in the article/supplementary material. Further inquiries can be directed to the corresponding author/s.

## Author contributions

PS designed, performed, and evaluated the experiments. JS, CR and RV helped the experiments. CK performed the statistical analysis. PS wrote the manuscript and JS, CK, SS, KY, and NS checked the manuscript. SR critically revised the manuscript. All authors contributed to the article and approved the submitted version.
